# Breeding objectives and trait prioritization in indigenous goat systems: insights from South African smallholders

**DOI:** 10.1007/s11250-026-05129-z

**Published:** 2026-06-12

**Authors:** Sibulele Praise Ntonga, Ziyanda Mpetile, Conference Thando Mpendulo, Oluwakamisi Festus Akinmoladun

**Affiliations:** 1 Department of Animal and Pasture Science, Faculty of Science and Agriculture, Private Bag X1314, Alice, Eastern Cape 5700 South Africa; 2 Department of Agricultural Economics and Animal Production, University of Limpopo, Turfloop Sovenga, Mankweng, 0727 South Africa; 3 Discipline of Agriculture and Food Technology, School of Agriculture, Geography, Environment, Ocean and Natural Sciences, The University of South Pacific, Samoa Campus, Apia, Samoa

**Keywords:** Breeding objectives, Trait preferences, Xhosa goats, Community-based breeding, Smallholder systems

## Abstract

Defining farmer-driven breeding objectives is essential for designing community-based breeding programs (CBBPs) that improve productivity and sustainability of indigenous goats. This study investigated herd structures, management practices, and trait preferences of 240 smallholder households keeping Xhosa goats across three agro-ecological zones (Savanna, Nama Karoo, and Grassland) in Eastern Cape Province, South Africa. Structured questionnaires were administered, and data analyzed using descriptive statistics, chi-square tests, ranking indices, and K-means cluster analysis. Results showed that goat ownership was predominantly male-headed (68%), with average herd sizes of 21.4 ± 5.7 goats. Breeding does comprised 54% of herds, while buck ownership was low (27%), raising concerns of inbreeding. The main reasons for keeping goats differed significantly across zones (χ² = 18.42, *p* = 0.016), with cultural uses ranked highest in Savanna, while meat and investment dominated in Nama Karoo and Grassland. Culling was primarily due to poor reproduction (31%), old age (26%), and disease (22%). Cluster analysis identified three typologies of goat keepers, traditional (41%), semi-modern (35%), and commercial-oriented (24%), that differed significantly in herd size (*p* < 0.01), mating control (*p* < 0.05), and trait prioritization. Trait ranking revealed that farmers prioritized fertility (Index = 0.241; Rank 1), maternal ability (0.192; Rank 2), and twinning ability (0.175; Rank 3) for does, while overall health (0.181; Rank 1), testes size (0.150; Rank 2), and reproductive soundness (0.156; Rank 1 in Ngqwara) were most important for bucks. Aesthetic traits such as coat colour (0.091–0.114) and horns (0.118) were consistently ranked lowest. This study demonstrates that farmers in communal systems emphasize reproductive, maternal, and adaptive traits over appearance or record-based criteria when selecting breeding stock. Aligning CBBPs with these farmer-defined priorities, while progressively integrating pedigree and performance data, is crucial for sustainable genetic improvement and resilience of indigenous goat populations in South Africa.

## Introduction

Goats are among the most important livestock species for smallholder households across sub-Saharan Africa, serving multiple functions including provision of meat, milk, hides, income, social status and, in many cases, cultural and ceremonial roles (van Wyk et al. 2022; Khowa et al. 2023). Their adaptability to harsh and variable environments, low input requirements, and rapid reproductive rates make them especially suited to resource‐limited farming systems (Akinmoladun et al. 2019, 2020a, b, 2023). In South Africa, the Eastern Cape Province harbors nearly 1.9 million goats, representing the country’s largest goat population. Nationally, smallholder farmers own approximately 5.1 million goats, with the Eastern Cape, Limpopo, and KwaZulu-Natal accounting for about 70% of the national herd (DAFF 2018; NAMC 2024).

Despite this potential, productivity in many villages or communal goat systems remains low, constrained by factors including poor management practices, inadequate health care, uncontrolled breeding, feed shortages, and weak market integration (Alemu 2020; Tada et al. 2025). Recent work shows that market price determinants for goats are heavily influenced by body size, colour, age, season, breed, and head type, and that farmers often sell in December to meet cultural and ceremonial demands (Mthi et al. 2024). Additionally, productivity traits such as birth weight are impacted by non-genetic factors (e.g., health status, weather extremes, nutrition of doe during pregnancy, mating control and buck availability etc.), in nationally dispersed goat breeds (Ramoroka et al. 2020).

Globally, there is increasing recognition that breeding program design must integrate farmer preferences and production objectives to be sustainable. Conventional centralized or government-led breeding initiatives have often failed in low-input systems because they overlook the realities of smallholder farmers (Haile et al. 2023). Community-based breeding programs (CBBPs) have emerged as a viable alternative, successfully implemented in Ethiopia, Tanzania, and other low-input systems, where participatory approaches ensured farmer ownership, reduced inbreeding, and improved genetic gains in small ruminants (Mirkena et al. 2012; Gizaw et al. 2021). However, while there is evidence from East Africa and parts of West Africa, relatively fewer studies in Southern Africa have systematically integrated farmer-defined breeding objectives with herd structure and management typologies, particularly in the context of genetic conservation of indigenous ecotypes.

In South Africa’s Eastern Cape Province, goats are a critical livelihood asset for rural and communal farmers. Moreover, previous studies showed that goat production is often characterized by small herd sizes, high mortality of young animals, skewed gender ratios of breeding stock, limited control of mating, and insufficient breeding buck ownership. For example, Kululeko (2015) found that in coastal areas of Eastern Cape, many households did not keep breeding bucks yet experienced high inbreeding levels and reduced fertility in their herds (Kululeko 2015). Similarly, in characterizations across multiple provinces, mean herd sizes in village goat systems are highly variable, with Eastern Cape households sometimes holding larger herds, but still suffering from constraints that limit productivity and impede improvement (Mdladla et al. 2017).

Trait preference and breeding practices have increasingly been recognized as key levers for designing CBBPs or other development interventions (Haile et al. 2023; Mathapo et al. 2025). Farmers’ preferences for traits such as fertility, maternal ability, growth rate, disease resistance, and prolificacy often influence which animals are selected for breeding, which in turn affect herd structure, kid recruitment, and overall herd dynamics (Chenyambuga and Lekule 2014; Tyasi et al. 2022; Richardson et al. 2023). A recent study in Lepelle-Nkumpi Local Municipality, for instance, found significant variation among villages in methods of controlling mating, sources of breeding bucks, reasons for culling, and culling methods, reflecting local adaptation, social norms, and resource constraints (Tyasi et al. 2022).

Despite a body of descriptive work, there are still gaps in understanding how herd structure, demography, mating control, and management typologies vary *within* and *between* agro-ecological zones, and how these differences translate into differences in production outcomes, kid survival, or genetic health. Also lacking in many studies is a thorough integration of preference and trait ranking data alongside demographic and herd‐structure data, to discern which practices are actually associated with better performance, and therefore plausible targets for extension or intervention.

The present study characterized goat herd structure, management practices, mating control, trait preferences, and cluster-based typologies among goat‐keeping households in three agro‐ecological regions of the Eastern Cape. Specifically, the study analyzed how counts of breeding and non‐breeding animals (does, bucks, castrates, kids), and practices such as mating control and buck ownership differ across regions; identified clusters/typologies of management style; and explored the preferences and ranking of traits by farmers. These insights are expected to contribute to more tailored breeding program design, improved kid survival, more efficient marketing, and enhanced livelihoods for smallholder goat keepers.

## Methodology

### Ethical clearance

Before the experiment, ethical consideration was applied for and obtained at the Inter-faculty Human Research Ethics Committee (IFHREC) in the University of Fort Hare (Ref. number: MPE021SNTO01).

### Study site and data collection

The study was conducted in the Eastern Cape Province of South Africa, focusing on three major agro-ecological zones, Savanna, Grassland, and Nama Karoo, as classified by Ndhleve et al. (2021). In the first stage, one representative location was selected from each zone, namely Sheshegu (Savanna), Ngqwara (Nama Karoo), and Ntshamanzi (Grassland). These locations were chosen because they host substantial populations of Xhosa goats and present contrasting ecological conditions that could influence phenotypic traits.

**Fig. 1:**
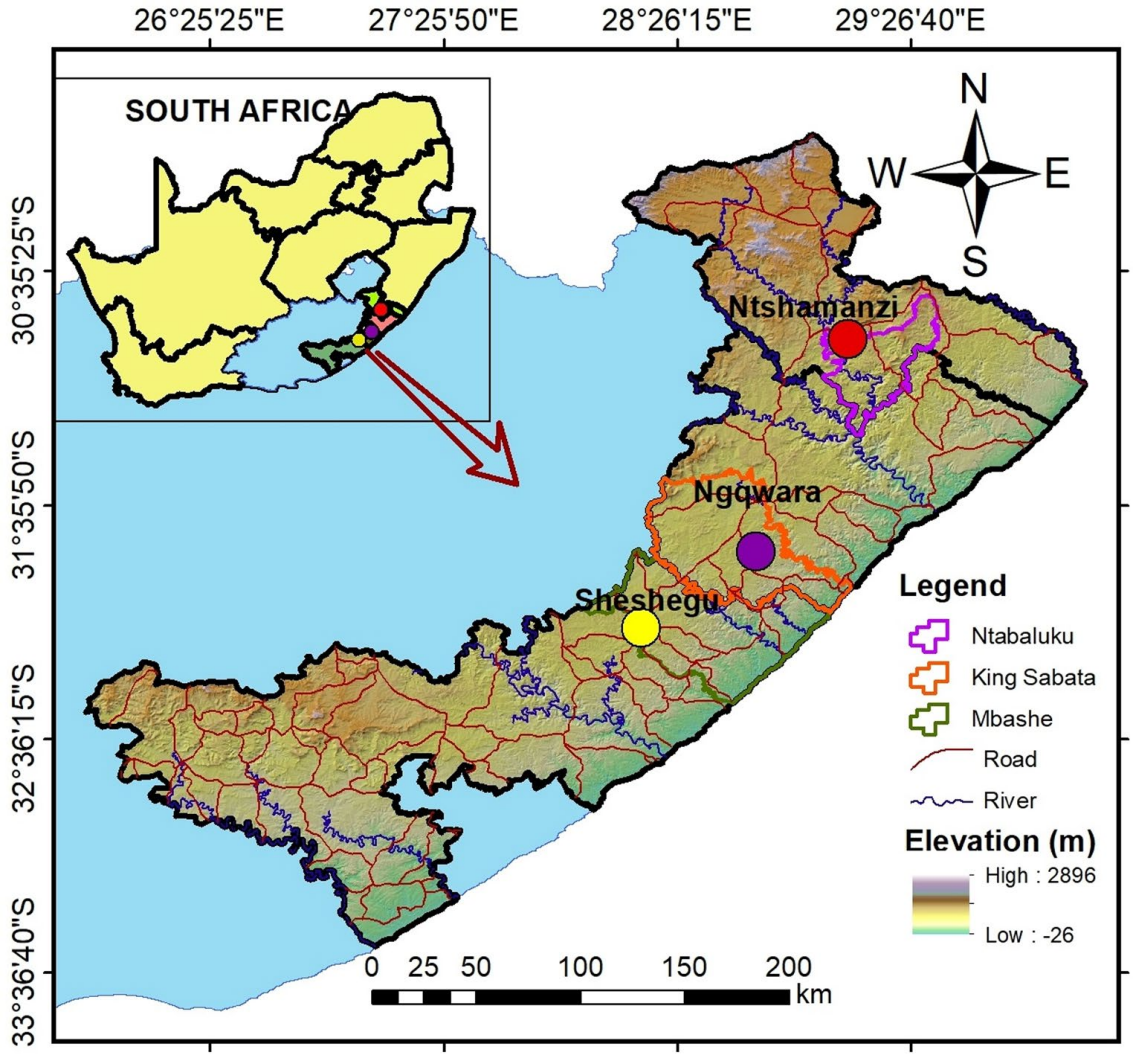
Location map of the sampling zones

The three zones differ markedly in climate, vegetation, and topography. Sheshegu (Savanna) receives moderate rainfall (450–600 mm annually), with July having the lowest average temperature of 6.3 °C and February having the highest average of 25 °C and is dominated by Bisho Thornveld vegetation consisting of thorny shrubs and scattered grasses adapted to semi-arid conditions. Positions of the area are 32°53′47′′58 S, 26°47′8′′E, and 544 m above sea level. Ngqwara (Nama Karoo) experiences an arid to semi-arid climate with an average annual rainfall of 469.9 mm, characterized by hilly terrain, rolling grasslands, deep gorges, and high diurnal temperature variation with hot days and cold nights. The positions in the area are 31°44’50.0"S, 28°44’03.0"E and 886 m above sea level. Ntshamanzi (Grassland), by contrast, records the highest annual rainfall (657 mm) and supports extensive grassveld interspersed with patches of coastal forest, providing relatively richer forage resources that may enhance growth and body condition. Ntshamanzi is located in 32.33627°S, 28.09574°E. These contrasting environmental profiles provided the scientific basis for selecting the three agro-ecological zones. The location map is shown in Fig. 1.

### Sampling and sample size determination

A multi-stage sampling strategy was used. First, the three agro-ecological zones were purposively selected based on their importance in indigenous Xhosa goat production. Within each zone, villages were randomly selected, and from these, households were randomly chosen from local farmer association lists and agricultural extension records. To ensure representation, proportional sampling was applied across the zones, yielding a total of 240 households (80 per zone). The sample size was determined using Cochran’s (1977) equation for categorical data:

$$\:n=\frac{{Z}^{2}\:p(1-p)}{{e}^{2}}$$

where Z is the Z-score at 95% confidence (1.96), p is the assumed proportion of households engaged in goat farming (0.8, based on provincial livestock statistics), and e is the margin of error (0.05). This yielded a minimum of 246 households; 240 households were finally surveyed after accounting for availability and consent, providing sufficient power (> 80%) to detect differences across zones.

### Questionnaire development and validation

The questionnaire was developed based on literature on smallholder goat production systems (e.g., Kosgey and Okeyo 2007; Gizaw et al. 2021). It covered household demographics, herd structure, breeding practices, trait preferences, and culling decisions.

To ensure validity and reliability, the instrument was pilot tested with 20 farmers outside the study zones, and modifications were made for clarity and contextual relevance. Internal consistency of trait preference items was assessed using Cronbach’s alpha (Cronbach 1951), which yielded a value of 0.82, indicating high reliability.

### Data collection and analysis

Data were collected by trained enumerators through face-to-face interviews in isiXhosa, the local language, to minimize misinterpretation. Farmers were asked to rank traits and breeding objectives according to importance, and demographic and herd data were cross-checked with direct observation where possible. Descriptive statistics (frequencies, percentages) were generated using SPSS v20 (IBM Corp 2011). Chi-square tests were used to examine associations between categorical variables (e.g., breeding practices across zones), and one-way ANOVA or Kruskal–Wallis tests were applied for continuous variables depending on normality.

Farmer preferences for breeding traits were analyzed using a ranking index method following Bett et al. (2009) and Zewdu et al. (2018). Farmers assigned ranks to traits (1 = most important, n = least important), which were then weighted using index formula.

$$\:Index=\:\frac{\sum\:\left({n}_{r}\:\times\:\:\left(n+1-r\right)\right)}{\sum\:\sum\:\left({n}_{r}\times\:\:\left(n+1-r\right)\right)}\:$$

where n_r_ is the number of responses for rank r and n is the maximum rank. The index values were used to generate relative importance scores, with higher values indicating stronger farmer preference. The ranking index approach used in this study captures the *relative importance of traits as perceived by farmers* and reflects ordinal preference rather than cardinal or quantitative weighting. Consequently, index values should not be interpreted as marginal utilities, economic weights, or direct genetic importance of traits. Rather, they provide a structured means of summarizing and comparing farmers’ subjective priorities across traits and locations.

K-means cluster analysis was used to identify typologies of goat-keeping households based on management and breeding-related variables. Variables included in the clustering procedure were: agro-ecological location, breeding season, ownership of a local breeding buck (yes/no), mating type (natural/uncontrolled, controlled, or artificial), mating control practices (yes/no), source of breeding buck, purpose of keeping a buck, ability to identify sires under uncontrolled mating (yes/no), reason for not controlling mating, goat selling season, and age at sale. Categorical variables were dummy-coded prior to analysis, while ordinal variables were coded using increasing integer values reflecting their inherent order. All variables were subsequently standardized to z-scores to remove scale effects and ensure equal contribution to the clustering solution. K-means clustering was conducted with multiple random starts to improve stability of solutions. The optimal number of clusters was determined by comparing internal validation indices, including silhouette width and the gap statistic. Cluster solutions were compared across two to five clusters, and the optimal three-cluster solution was determined using average silhouette width and biological interpretability of the resulting clusters. To assess cluster distinctiveness, one-way ANOVA was applied to identify variables that significantly differentiated clusters. Clusters were subsequently interpreted and labelled based on dominant management and breeding characteristics. All statistical tests were conducted at a significance threshold of *p* < 0.05.

## Results and discussion

### Socio-demographics and herd structure

The cross-tabulation of agro-ecological zones with demographic characteristics, breeding practices, and herd structure is presented in Tables 1 and 2. Across Savanna, Nama Karoo, and Grassland zones, most Xhosa goat keepers were middle-aged (41–60 years), from male-headed households, and had large family sizes (> 5 members). Education levels varied, with many respondents having only primary or no formal education, consistent with patterns reported for communal livestock keepers in South Africa and sub-Saharan Africa (Mdladla et al. 2017; Rumosa-Gwaze et al. 2009). While marital status did not differ by zone, both farming experience and the purpose of keeping bucks were significantly associated with agro-ecological region (*p* < 0.05), indicating context-specific production objectives.

Mating was predominantly natural and uncontrolled across all zones, with limited use of controlled or artificial mating. Although some farmers reported the ability to identify sires under communal grazing conditions, this practice was inconsistent, reflecting the practical constraints of communal systems (Malenje 2023; Tyasi et al. 2022). Limited sourcing of breeding bucks from outside the community further raises concerns about inbreeding and reduced genetic diversity, as previously documented in communal goat systems (Kululeko 2015). These findings suggest that demographic factors and entrenched management practices may constrain the adoption of structured breeding programs, highlighting the need for targeted extension and training interventions.

**Table 1:** Demographic characteristics and breeding practices according to location

Variables	Groups	Locations* (%)			χ	*P*-value
		Savanna (%)	Nama Karoo (%)	Grassland (%)		
Gender	Male Female	82.5 17.5	82.5 17.5	65 35	4.565	0.102
Age	21–40 41–60 > 60	20 50 30	10 47.5 42.5	7.5 45 47.5	4.530	0.339
Family size	< 5 > 5	45 55	42.5 57.5	37.5 62.5	0.480	0.787
Highest education	Primary Secondary Tertiary None	20 32.5 25 22.5	22.5 35 30 12.5	15 37.5 32.5 15	2.452	0.874
Marital status	Married Single Widow	57.5 32.5 7.5	70 20 10	65 22.5 10	4.302	0.829
Work experience	< 5 6–10 11–15 > 15	17.5 35 27.5 20	5 15 47.5 32.5	0 15 42.5 42.5	19.01	0.004
Breeding season	Winter Autumn Spring Summer	22.5 15 5 57.5	27.5 5 7.5 60	20 2.5 10 67.5	6.185	0.403
Main reason for keeping goats	Cultural/traditional Investment Meat Milk Manure	35 25 22.5 10 7.5	22.5 32.5 27.5 10 7.5	20 35 30 8.75 6.25	18.42	0.016
Do you have local bucks?	Yes No	67.5 32.5	55 45	42.5 57.5	5.051	0.080
Does your buck serve other does?	Yes No	70 30	62.5 37.5	70 30	0.684	0.710
Does your does serve other bucks?	Yes No	52.5 47.5	62.5 37.5	75 25	4.378	0.112
What is the mating system adopted?	Natural/uncontrolled Artificial Controlled	77.5 5 17.5	62.5 0 35	75 2.5 22.5	5.083	0.279
If uncontrolled, could you identify the sire of a kid?	Yes No	65 35	70 30	67.5 32.5	0.228	0.892
Method of controlling mating	Castration Culling Buck-doe separation	60 10 30	65 17.5 17.5	65 10 25	10.84	0.093
Reason for not controlling mating	Goats graze together Ignorance both	65 17.5 17.5	62.5 22.5 15	75 15 10	1.978	0.740
Source of breeding buck	Born in the herd Outside the farm purchase Purchase or borrowed from a friend	37.5 40 22.5	62.5 17.5 20	65 20 15	8.682	0.070
Purpose of keeping a buck	Mating only Fattening only Both	27.5 5 67.5	40 5 55	65 2.5 32.5	11.87	0.018
At what age (yrs) do you sell your goats?	< 2 2–5 > 5	22.5 72.5 5	17.5 67.5 15	25 60 15	7.043	0.721
Which season do you sell your goats?	Summer Winter Autumn Spring All year round	40 7.3 15 5 32.5	47.5 12.5 2.5 12.5 25	50 22.5 10 0 17.5	14.45	0.071

*: 80 respondents for each geographical location

Herd structure varied across agro-ecological zones (Table 2), indicating differences in reproductive performance and management. Breeding does dominated herd composition, averaging 12.20–13.65 animals per household, while breeding bucks were few (0.43–0.77), resulting in very low buck-to-doe ratios. Castrates were more common in Savanna (mean 2.36) than in Grassland (1.90) and Nama Karoo (1.15). Kids under five months were most numerous in Grassland (mean 6.18), followed by Savanna (5.43) and Nama Karoo (4.85). Overall herd sizes were moderate and heavily skewed toward does and young stock.

**Table 2:** Mean herd structure of Xhosa goats across the three agroecological zones

Regions		Breeding doe (> 1 year)	Breeding buck (> 1 year)	Castrates	Buck kids (5 months- 1 year)	Doe kids months- 1 year)	Kids (< 5 months)
Savanna	Mean SD	12.20 4.28	0.75 0.67	4.52 1.60	2.90 1.42	3.97 1.99	4.72 2.14
Nama Karoo	Mean SD	12.42 4.71	0.65 0.70	4.50 1.32	2.85 1.38	3.40 1.79	4.73 2.19
Grassland	Mean SD	13.65 4.33	0.77 0.83	3.60 1.83	3.07 1.43	3.80 1.60	6.18 2.38
	P-values	0.050	0.012	0.023	0.011	0.010	0.000

The dominance of breeding does and scarcity of bucks aligns with patterns in communal goat systems, where does are retained for reproduction and bucks are often sold or culled (Rumosa-Gwaze et al. 2009; Nguluma et al. 2020). However, the very low number of breeding bucks is unlikely to meet recommended buck-to-doe ratios (1:25–1:35), increasing the risk of inbreeding under uncontrolled mating systems (Kosgey 2004; Kululeko 2015). Higher kid numbers in the Grassland zone may reflect more favorable feed availability, management, or health conditions, consistent with reports from other African agro-ecological settings (Chenyambuga and Lekule 2014; Abebe et al. 2020). The greater presence of castrates in Savanna and Nama Karoo likely reflects management strategies related to herd control, marketing, or cultural uses, as noted by Mtshali et al. (2021).

### Cluster and typology analysis

The cluster analysis results of farmers’ breeding practices and summary of ANOVA for the cluster analysis are shown in Tables 3a and 3b, respectively. The three-cluster solution showed acceptable separation based on average silhouette width. Respondents in Cluster 1 (traditional goat keepers) exhibited characteristics aligned with traditional, low-input goat production systems. These farmers showed a relatively high reliance on local breeding bucks (z = 0.55) and engaged less in structured mating practices, as evidenced by a negative z-score for mating type (z = -0.37) and moderate mating control (z = 0.28). Although formal mating control was limited, they showed above-average ability to identify sires even under uncontrolled mating conditions (z = 0.34), suggesting a level of observational knowledge and familiarity with individual animals, common in small, closely managed herds. Notably, this group strongly cited environmental or communal grazing constraints as the main reason for not controlling mating (z = 1.43), consistent with findings in communal systems where herds graze freely.

**Table 3a:** Final cluster centers (Z-scores)

Variable	Cluster 1^a^ (*n* = 58)	Cluster 2^b^ (*n* = 88)	Cluster 3^c^ (*n* = 94)
Location	-0.25	0.47	-0.29
Breeding season	0.18	0.30	-0.39
Do you have local buck?	0.55	0.01	-0.35
Mating type	-0.37	0.78	-0.51
Mating control	0.28	0.40	-0.55
Goat selling season	0.20	-0.09	-0.03
Goat selling age	-0.24	-0.01	0.16
Purpose of keeping buck	-0.29	-0.31	0.47
Source of breeding buck	-0.11	-0.35	0.40
Can you identify the sire if mating is uncontrolled	0.34	-0.50	0.26
Reason for Not Controlling Mating	1.43	-0.39	-0.52

^a^Traditional keepers; ^b^Semi-modern keepers; ^c^Commercial-oriented farmers; n = No. of cases

**Table 3b:** ANOVA summary (cluster analysis)

Variable	F-value	*p*-value
Reason for mating not controlled	115.71	0.000
Mating type	32.87	0.000
Mating control	14.31	0.000
Purpose of keeping buck	9.61	0.000
Can you identify the sire if mating is uncontrolled	9.96	0.000
Do you have local buck	8.05	0.001
Breeding buck source	7.46	0.001
Breeding season	6.61	0.002
Selling goat season	0.77	0.464
Goat selling age	1.46	0.236

Cluster 2 (semi-modern keepers) represents farmers transitioning towards more organized production systems. These respondents recorded the highest z-scores for both mating type (z = 0.78) and mating control (z = 0.40), suggesting more formalized breeding practices compared to the other clusters. Although ownership of local bucks was moderate (z = 0.01), their sourcing patterns (z = -0.35) and buck-keeping purpose (z = -0.31) suggest limited emphasis on buck selection or investment in breeding males. Interestingly, this group scored negatively regarding their ability to identify sires under uncontrolled mating (z = -0.50), indicating possible gaps in monitoring individual matings despite structural efforts. Their reported reasons for not controlling mating were minimal (z = -0.39), implying awareness of and efforts to manage breeding activity. This group may represent farmers with increased access to extension services or exposure to semi-commercial production practices without fully integrated management systems. Their responses suggest a willingness to adopt improved practices, but resources or knowledge constraints may still exist.

Cluster 3 (commercial-oriented farmers) is characterized by more strategic and economically motivated production practices. These farmers showed above-average values in buck-keeping purpose (z = 0.47) and sourcing (z = 0.40), indicating a clear intention to rear and use bucks for mating and fattening, a hallmark of dual-purpose commercial systems. Although they had below-average control over mating (z = -0.55) and mating type (z = -0.51), this group did not cite significant constraints for not controlling mating (z = -0.52), suggesting their practices may be driven more by choice or scale than by lack of knowledge. Their slightly above-average age at sale (z = 0.16) supports the idea of holding animals longer for fattening or higher market value. They scored negatively on local buck ownership (z = -0.35), likely due to intentional crossbreeding or sourcing bucks externally for genetic improvement. The profile of this group suggests a shift towards commercial viability, aligning with market-responsive breeding strategies observed in peri-urban and emerging farmer contexts.

The clustering results highlight the heterogeneity of goat management systems within the same province. This heterogeneity mirrors findings in other African contexts, such as Ethiopia (Haile et al. 2023) and Tanzania (Chenyambuga and Lekule 2014), where farmers group into typologies vary in resource endowment, breeding practices, and productivity outcomes. Similar studies in West Africa (Badjibassa et al. 2024) have also documented clusters of farmers who either rely exclusively on home-born bucks or selectively introduce external sires, leading to divergent reproductive outcomes.

The practices observed among Cluster 1 households suggest a degree of informal yet consistent breeding awareness, characterized by reliance on local bucks, observational sire identification, and selective retention. By contrast, the low inferential response of Cluster 2 emphasizes the vulnerabilities of households with small herd sizes and limited access to breeding bucks. These households may serve as priority targets for CBBPs, where cooperative buck sharing or rotational schemes can mitigate inbreeding and improve reproductive efficiency (Kosgey and Okeyo 2007; Haile et al. 2023). Comparatively, the intermediate strategy observed in Cluster 3 resembles transitional systems reported in Burkina Faso and Mali, where some farmers blend traditional and semi-structured practices in response to changing markets (Badjibassa et al. 2024). This suggests that as markets deepen, more households may shift from purely subsistence-oriented strategies toward integrated approaches that combine productivity and cultural objectives.

## Ranking analysis

### The purpose of keeping goats and reasons for culling them

The purpose of keeping goat by communal farmers as well as the reason for their culling in various locations is represented in Table 4. While farmers in Savanna primarily kept goats for traditional purposes, farmers in Nama Karoo and Grassland kept goat primarily for investments. Meat production alternates between investment and traditional purposes across the three provinces. Manure use and milk production were generally ranked lowest (Ranks 4–5), reflecting their relatively minor role in these systems. These findings affirm the multifunctional role of goats in communal production systems, where they provide both tangible (meat, income) and intangible (cultural, social) benefits.

**Table 4:** The purpose of keeping goat and their reason for culling

Variables	Savanna					Nama Karoo					Grassland				
	R1	R2	R3	Index	Rating	R1	R2	R3	Index	Rating	R1	R2	R3	Index	Rating
*Goat keeping purpose*															
Investment	13	15	12	0.218	3	9	24	7	0.217	1	12	12	16	0.233	1
Meat production	9	22	9	0.220	2	15	15	10	0.208	3	14	20	6	0.200	3
Milk Production	20	14	6	0.182	5	24	8	8	0.178	5	21	14	5	0.178	5
Manure	20	13	7	0.186	4	20	14	6	0.183	4	18	18	4	0.183	4
Traditional purposes	17	15	8	0.366	1	12	19	9	0.214	2	15	16	9	0.208	2
*Reason for culling goats*															
Old Age	9	8	23	0.207	3	10	14	16	0.202	3	13	10	17	0.196	3
Sickness	7	10	23	0.211	2	13	13	14	0.191	4	9	13	18	0.208	2
Reproductive problem	5	10	25	0.220	1	8	10	22	0.221	1	4	17	19	0.222	1
Physical defect	7	16	17	0.198	4	7	19	14	0.205	2	9	13	18	0.208	2
Unwanted physical appearance	19	8	13	0.163	5	15	13	12	0.181	5	20	9	11	0.166	4

Previous studies have highlighted similar patterns, with goats acting as “multi-purpose assets” for rural households in South Africa and beyond (Peacock 2005).

The prominence of cultural use underscores the embeddedness of goats in social identity and customary practices, echoing findings from Eastern Cape and Limpopo where goats are indispensable for rituals, initiation ceremonies, and dowry payments (Mdladla et al. 2017; Tyasi et al. 2022). The listing of goats for meat production in the top three priorities reflects the central role of goats in household food security. This finding is consistent with studies in Ethiopia and Tanzania where meat is the dominant production objective (Abebe et al. 2020; Chenyambuga and Lekule 2014). Differences across zones also suggest that breeding and management interventions must be sensitive to cultural drivers. For instance, interventions aimed solely at maximizing meat production may overlook farmers’ strong preferences for traits associated with cultural suitability, such as coat color or horn size (Chenyambuga and Lekule 2014). Recognizing these broader objectives is crucial for designing CBBPs that are socially acceptable and sustainable (Haile et al. 2023).

Reproductive problems were ranked as the primary reason for culling across the three zones. Old age and physical defects closely followed as reasons for culling goats in the zones surveyed. Culling due to reproductive inefficiency underscores farmers’ recognition that fertility is central to productivity. Comparable findings have been reported in Ethiopia, where infertility was the top reason for culling breeding females (Abebe et al. 2020). Old age as the second most common reason is logical in resource-constrained systems, as unproductive aged animals consume scarce resources without contributing to offspring. Physical defects and sometimes disease-related culling indicate awareness of the economic cost of morbidity, consistent with patterns in communal herds across Southern Africa. Lower emphasis on physical defects suggests that while body size is important for selection, it is not the primary basis for removing animals once they enter the herd (Sheriff et al. 2021).

### Visual and functional selection of breeding bucks according to rank

The ranking of traits used for selecting breeding bucks across agro-ecological zones is presented in Table 5. Overall health ranked highest in all zones, followed by testes size and body size, although minor variations occurred across locations. Functional traits, including reproductive soundness, disease resistance, and adaptability, consistently received higher index values than aesthetic traits. Coat colour, horns, and temperament were ranked lowest across all zones.

The consistent prioritization of health and testes size reflects farmers’ reliance on easily observable indicators of reproductive fitness and survival under low-input communal systems. Disease resistance and adaptability were particularly emphasized in Savanna and Grassland zones, where veterinary support is limited and grazing conditions are variable. Reproductive soundness ranked highest among functional traits in Nama Karoo and Grassland, underscoring the centrality of fertility in buck selection.

Pedigree information received moderate ranking, particularly in Nama Karoo, suggesting some recognition of lineage quality despite the absence of formal record-keeping. Performance records and temperament were consistently ranked low, reflecting practical constraints in communal systems. Overall, the results indicate a strong farmer preference for functional and adaptive traits over appearance-based characteristics, consistent with findings from communal goat systems in southern and eastern Africa (Mdladla et al. 2017; Monau et al. 2020; Haile et al. 2023).

**Table 5:** Visual and functional selection criteria for breeding buck

Variables	Savanna					Nama Karoo					Grassland				
	R1	R2	R3	Index	Rating	R1	R2	R3	Index	Rating	R1	R2	R3	Index	Rating
*Visual appearance of breeding buck*															
Liveliness/Aggressiveness	4	13	23	0.154	2	10	14	16	0.141	5	10	15	15	0.141	5
Presence of horns	12	12	16	0.131	5	9	19	12	0.136	6	7	17	16	0.148	4
Testes size	4	14	22	0.153	3	7	15	18	0.149	2	8	14	18	0.150	2
Legs and feet	7	15	18	0.142	4	8	14	18	0.148	3	12	14	14	0.136	6
Color	20	8	12	0.112	7	15	12	13	0.128	7	16	15	9	0.121	7
Body Size	4	0	26	0.128	6	12	8	20	0.144	4	8	14	18	0.150	2
Overall health	1	2	37	0.181	1	9	9	22	0.153	1	9	10	21	0.153	1
*Criteria for breeding buck selection*															
Adaptability	2	8	30	0.153	2	9	13	18	0.141	5	5	16	19	0.145	3
Disease resistance	1	3	36	0.163	1	9	7	24	0.151	4	6	10	24	0.151	2
Reproductive soundness	6	13	21	0.135	5	5	12	23	0.156	1	7	7	26	0.153	1
Longevity/ Production efficiency	7	12	21	0.133	6	5	14	21	0.152	2	6	16	18	0.142	5
Genetic pedigree	5	6	29	0.147	3	6	12	22	0.152	2	8	16	16	0.136	6
Performance records	5	9	26	0.143	4	15	14	11	0.121	7	9	19	12	0.128	7
Temperament/behavior	6	19	15	0.126	7	8	18	12	0.127	6	6	15	19	0.144	4

### Visual and functional selection of breeding does according to rank

The ranking of selection criteria for breeding does is shown in Table 6. Fertility was consistently ranked as the most important trait across all agro-ecological zones, followed by maternal ability, twinning ability, and body size. Disease resistance, temperament, and coat colour were ranked lowest.

The dominance of fertility highlights farmers’ prioritization of reproductive efficiency as the foundation of herd productivity. Maternal ability and twinning ability ranked closely behind fertility, reflecting the importance of kid survival and herd growth in communal systems. Visual reproductive indicators such as the teat and udder size were also prioritized, reinforcing the focus on maternal performance.

Body condition was particularly emphasized in the Grassland zone, where it ranked highest, indicating the use of visible health and nutritional status as a proxy for reproductive readiness. In contrast, aesthetic traits such as coat colour and topline/rump were consistently deprioritized across all zones, suggesting that reproductive and survival traits outweigh appearance in doe selection decisions. Similar patterns have been reported among goat keepers in South Africa, Uganda, and Ethiopia (Onzima et al. 2018; Gizaw et al. 2011; Tilahun et al. 2023).

While ranking indices effectively capture farmer preferences, they do not quantify genetic or economic responses to selection. Translation of these priorities into formal breeding objectives would require complementary genetic and bio-economic analyses. To enhance the applied relevance of these findings, future work should focus on translating farmer-prioritized traits into measurable and operational selection criteria suitable for low-input systems. For example, fertility preferences could be proxied using kidding interval or number of kids weaned per doe per year, while maternal ability may be reflected through kid survival rates and early growth performance.

**Table 6:** Visual and functional selection criteria for selection breeding does

Variables	Savanna					Nama Karoo					Grassland				
	R1	R2	R3	Index	Rating	R1	R2	R3	Index	Rating	R1	R2	R3	Index	Rating
*Visual selection for breeding does*															
Udder size	1	11	28	0.144	3	11	12	17	0.124	5	6	14	20	0.132	3
Teat conformation	5	10	25	0.235	1	5	18	17	0.133	2	6	17	17	0.128	4
Body condition	1	5	34	0.152	2	9	13	18	0.128	4	5	13	22	0.136	1
Color	15	16	9	0.099	8	14	13	13	0.114	8	15	11	14	0.111	7
Top line and rump	8	23	9	0.109	7	8	22	10	0.118	6	13	16	11	0.109	8
Feet and legs	8	15	17	0.120	5	8	11	21	0.134	1	9	15	16	0.122	6
Temperament	8	20	12	0.113	6	12	14	14	0.118	6	3	24	13	0.126	5
Overall soundness	7	10	23	0.129	4	8	14	18	0.130	3	6	11	23	0.136	1
*Selection criteria for breeding does*															
Disease resistance	2	4	34	0.140	1	7	9	24	0.136	2	6	11	23	0.136	2
Fertility	5	2	33	0.138	2	6	8	26	0.140	1	8	4	28	0.140	1
Maternal Ability	3	10	27	0.130	3	7	9	24	0.136	2	7	15	18	0.127	4
Twinning ability	6	18	16	0.112	7	11	13	16	0.120	4	12	17	11	0.110	8
Age at first Kidding	5	22	13	0.110	8	10	17	13	0.116	7	9	14	17	0.123	5
Body size	9	11	20	0.113	6	14	8	18	0.118	6	11	17	12	0.113	7
Longevity	4	12	24	0.125	4	11	15	14	0.116	7	7	23	10	0.116	6
Adaptability	1	10	29	0.125	4	12	10	18	0.120	4	5	14	21	0.134	3

Similarly, disease resistance and adaptability could be monitored using simple health records, frequency of treatment, or body condition scoring. The development of simplified, community-level recording schemes, implemented through participatory approaches, would allow farmers to systematically track these indicators over time. Such participatory performance monitoring frameworks have the potential to bridge the gap between subjective trait preferences and objective selection decisions, thereby supporting the gradual integration of data-driven breeding strategies within community-based breeding programs.

### Study limitations

This study provides important insights into herd structure, breeding practices, and farmer-defined trait preferences in indigenous goat systems; however, several limitations should be acknowledged.

First, the study did not include objective productivity indicators such as kidding rate, pre-weaning mortality, inter-kidding interval, growth performance, or offtake rate. Consequently, while management typologies were identified, it was not possible to empirically establish direct relationships between these typologies and measurable productivity outcomes. Future studies integrating longitudinal performance data would be necessary to validate the productivity implications of the identified clusters.

Second, the cross-sectional design of the study limits the ability to infer temporal changes or causal relationships in breeding practices and herd dynamics. Data were largely based on farmer self-reports, which may be subject to recall bias, particularly for events such as culling or reproductive performance. In addition, responses may have been influenced by social desirability bias, where farmers report practices perceived as favorable. Despite efforts to minimize these effects through trained enumerators and cross-verification with on-farm observations, these potential sources of bias should be considered when interpreting the findings.

## Conclusion

This study demonstrates that breeding objectives and trait preferences of Xhosa goat keepers in South Africa are primarily driven by livelihood demands, cultural values, and environmental constraints. Farmers emphasized reproductive and maternal traits in does and health, fertility, and adaptive traits in bucks, reflecting pragmatic strategies to sustain herd survival and productivity under low-input communal systems. The identification of distinct management typologies through cluster analysis highlights the diversity of production contexts and the necessity for flexible, context-specific breeding interventions. The low priority given to aesthetic and record-based traits further reveals a disconnect between conventional breeding program goals and farmer practices.

For effective and sustainable genetic improvement, community-based breeding programs (CBBPs) must be grounded in farmer-preferred functional traits while progressively translating these preferences into measurable and selectable indicators. While traits such as fertility, maternal ability, and disease resistance were consistently prioritized by farmers, their integration into breeding programs requires the development of simple, field-applicable proxies (e.g., kidding interval, kid survival rate, body condition scoring, and basic health records) that can be routinely monitored under communal conditions.

In this context, participatory approaches should be used to co-develop selection criteria that align farmer perceptions with objective performance indicators, thereby improving both adoption and genetic progress. The establishment of community-level recording schemes, even at a basic level, would enable the gradual incorporation of pedigree and performance data into selection decisions. Over time, these data can support more structured breeding strategies, including controlled buck rotation, within-community sire referencing, and the identification of superior breeding stock.

Three practical design implications emerge: (i) the introduction of controlled buck rotation or communal buck-sharing schemes to mitigate inbreeding under uncontrolled mating; (ii) the operationalization of farmer-preferred traits into measurable selection criteria supported by simple recording systems; and (iii) the use of farmer typologies to guide targeted entry points for training, extension, and breeding program design across different production systems.

Aligning genetic improvement objectives with farmer priorities and local management realities is essential for improving adoption, enhancing kid survival, and ensuring the sustainable use of indigenous goat genetic resources in South Africa’s smallholder systems.

## Data Availability

Dataset available upon reasonable request.
